# Avidin grafted dextran nanostructure enables a month-long intra-discal retention

**DOI:** 10.1038/s41598-020-68351-1

**Published:** 2020-07-21

**Authors:** Erica K. Wagner, Armin Vedadghavami, Timothy D. Jacobsen, Shakti A. Goel, Nadeen O. Chahine, Ambika G. Bajpayee

**Affiliations:** 1 Department of Bioengineering, Northeastern University, 805 Columbus Avenue, Boston, MA 02120 USA; 2 Department of Orthopedic Surgery, Columbia University, 650 West 168th Street, 14-1410, New York, NY 10032 USA; 3 Department of Biomedical Engineering, Columbia University, New York, NY USA; 4 Department of Orthopedic Surgery, Indian Spinal Injuries Center, New Delhi, India; 5 Department of Mechanical Engineering, Northeastern University, 360 Huntington Avenue, Boston, MA 02115 USA

**Keywords:** Biophysics, Permeation and transport, Biotechnology, Biomaterials, Nanobiotechnology

## Abstract

Low back pain is often the direct result of degeneration of the intervertebral disc. A wide range of therapeutics including anti-catabolic, pro-anabolic factors and chemo-attractants that can stimulate resident cells and recruit endogenous progenitors are under consideration. The avascular nature and the dense matrix of this tissue make it challenging for systemically administered drugs to reach their target cells inside the nucleus pulposus (NP), the central gelatinous region of the intervertebral disc (IVD). Therefore, local intra-discal injection of therapeutic drugs directly into the NP is a clinically relevant delivery approach, however, suffers from rapid and wide diffusion outside the injection site resulting in short lived benefits while causing systemic toxicity. NP has a high negative fixed charge density due to the presence of negatively charged aggrecan glycosaminoglycans that provide swelling pressures, compressive stiffness and hydration to the tissue. This negative fixed charge density can also be used for enhancing intra-NP residence time of therapeutic drugs. Here we design positively charged Avidin grafted branched Dextran nanostructures that utilize long-range binding effects of electrostatic interactions to bind with the intra-NP negatively charged groups. The binding is strong enough to enable a month-long retention of cationic nanostructures within the NP following intra-discal administration, yet weak and reversible to allow movement to reach cells dispersed throughout the tissue. The branched carrier has multiple sites for drug conjugation and can reduce the need for multiple injections of high drug doses and minimize associated side-effects, paving the way for effective clinical translation of potential therapeutics for treatment of low back pain and disc degeneration.

## Introduction

Low back pain (LBP) is the leading cause of disability worldwide, and its prevalence as well as burden increases with age^1^. It is estimated that 70–80% of the population will experience LBP at some point in their lives^2^, resulting in nearly $90 billion in annual costs in the United States for treatment^3^. LBP is commonly associated with intervertebral disc (IVD) degeneration (DD)^4^. The IVD is avascular and functions as a complex structure between vertebrae that transmits load and complex location, such as bending and twisting. The IVD is composed of the nucleus pulposus (NP), the gelatinous center region of the IVD, the annulus fibrosus, the tensile connective tissue that restricts the NP using circumferential, or hoop, stresses, and the cartilaginous endplate which connects the NP and annulus fibrosus to the vertebra^5^. Furthermore, the NP is densely composed of proteoglycans which have covalently attached anionic glycosaminoglycans (GAGs) resulting in a high negative fixed charge density of the NP (− 140 mM)^6–8^. These negatively charged groups are critical to the structure and function of the tissue by providing the needed swelling pressures, compressive stiffness, and hydration^7,9,10^. The early stages of DD are often characterized by a loss of extracellular matrix (ECM) GAGs resulting in decreased disc height and hydration, which alters biomechanical function^11^.

A wide range of therapeutics including anti-catabolic, pro-anabolic factors and chemo-attractants that can stimulate the resident NP cells and recruit endogenous progenitors are under consideration to treat DD^12^. The avascular nature of NP makes it challenging for systemically administered drugs to reach their target cells. Therefore, local intra-discal injection of therapeutic drugs or cells directly into the NP are the most clinically relevant delivery approach, especially since the NP tissue is well encapsulated by the endplate and annulus fibrosus. Drugs injected in this manner, however, suffer from rapid and wide diffusion outside the injection site resulting in short-lived benefits^13,14^. Toxicity concerns are further aggravated when potent biologics, gene editing/silencing vectors, or proteolytic enzymes are used^15–17^. Current in vitro and in vivo work relies on frequent drug dosing, which is clinically infeasible. It is, therefore, critical to develop effective approaches for enhancing intra-NP drug residence time and cell targeting.

Drug delivery systems under investigation include both nano- and micro-sized polymeric particles such as injectable microspheres, hydrogels, and tissue engineering scaffolds^18–24^ but have shown short intradiscal residence time of 24–48 h requiring the need for multiple injections^24^. Other systems consist of synthetic biodegradable polymers like poly(lactide) (PLA), poly(glycolide) (PGA), poly(ε-caprolactone) (PCL) for their tunable properties and minimal immunogenicity^25^. Despite these advances, there remains a need to develop a delivery system that can enable long-term retention of intra-discal injected drugs to maximize therapeutic benefits for patients and to minimize detrimental side effects.

The high negative fixed charge density of NP due to its dense network of negatively charged proteoglycans presents an opportunity for enhancing the residence time of cationic drug depots enabling sustained therapeutic benefits^26–28^. In similar highly negative tissues such as cartilage, a cationic glycoprotein Avidin (Av), due to its optimal size and net charge (7 nm diameter (dia), net charge between + 6 and + 20 estimated using Donnan–Boltzmann)^29,30^, was shown to penetrate through full thickness of rabbit knee cartilage following intra-articular injection^31^ resulting in a high intra-cartilage uptake ratio of 180 (implying 180 × higher concentration of Av inside cartilage than surrounding fluid at equilibration). Avidin was still found to be present through the full thickness of rabbit cartilage 2 weeks following its administration in vivo^31,32^. Recently, multi-arm Av nano-constructs were synthesized with high drug loading content^33,34^. When conjugated to Dexamethasone and administered in a single low dose 1 week following anterior cruciate ligament transection in a rabbit model, it suppressed injury induced joint inflammation, synovitis, incidence of osteophyte formation and restored trabecular properties significantly greater than free dexamethasone^32,35^. Based on Av’s structure, short length cationic peptide carriers have been used with similar charge range to penetrate full thickness articular cartilage via weak-reversible binding with negatively charged GAGs. This approach resulted in 100–400 times higher intra-cartilage concentrations compared to the neutral versions due to high Donnan partitioning factors^36^. Despite weak binding, the high negative fixed charge density of aggrecan associated GAGs inside tissues greatly increased their residence time.

**Figure 1:**
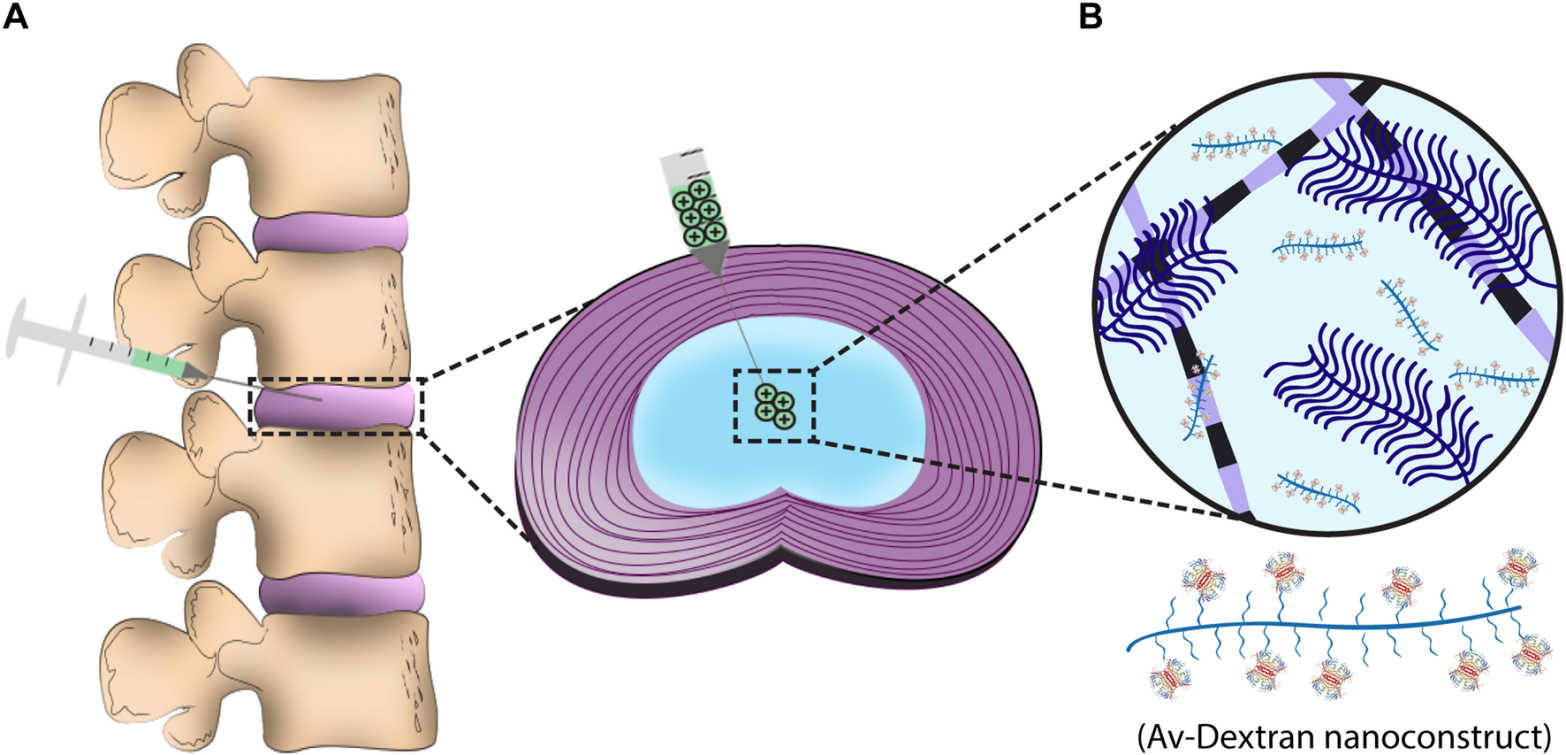
Charge based intra-discal drug delivery. (**A**) Direct injection to the nucleus pulposus (NP) to treat inflammation in the early stages of disc degeneration. (**B**) Intra-NP administered cationic Avidin grafted Dextran (Av-Dextran) nanostructure binds with negatively charged glycosaminoglycans resulting in a month-long intra-disc residence time owing to synergistic effects of electrostatic interactions and larger carrier size.

The goal of this study is to develop a cationic drug delivery system for intra-discal injection. Owing to differences in the biochemistry and biophysics of NP and articular cartilage ECM, where ratio of sGAG-to-collagen is an order of magnitude greater in NP compared to articular cartilage^37^, we investigate the intra-NP transport and binding properties of solutes as a function of charge and size. Since NP has similar net fixed charge density as cartilage^38^ but 10–100 × larger pore size^39^, we graft Av on 500 kDa Dextran to synthesize cationic nanostructures that combine synergistic effects of cationic charge and larger carrier size to sufficiently slow down intra-NP diffusivities due to electrostatic binding and steric hindrances, enabling a month long intra-NP residence time following intra-discal administration (Fig. 1). Studies were performed using bovine NP explants, a commonly used model of human IVD due to similarities in anatomical size, ECM content and cellularity. Since GAG loss can also significantly affect the retention and transport of drugs and drug carriers, we compare the transport and retention properties of cationic drug delivery system in GAG depleted explants to determine the effect of degeneration on optimization of drug delivery.

## Materials and methods

Agarose, low electroendosmosis was purchased from Alfa Aesar (Ward Hill, MA, USA). Av, FITC conjugate, Av, Texas Red conjugate, NeutrAvidin (Nu), FITC conjugate, Nu, Texas Red conjugate, Calcein AM, and Ethidium Homodimer-1 were purchased from Invitrogen (Eugene, OR, USA). Protease Inhibitor Mini Tablets, EDC (1-ethyl-3-(3-dimethylaminopropyl) carbodiimide hydrochloride), Sulfo-NHS (*N*-hydroxysulfosuccinimide), Quant-iT PicoGreen dsDNA Assay Kit, and Dimethyl Sulfoxide (DMSO) were purchased from Thermo Scientific Pierce (Rockford, IL, USA). Trypsin–EDTA phenol red, high glucose DMEM, and Pen-Strep Antibiotic–Antimycotic were purchased from Gibco (Carlsbad, CA, USA). Proteinase-K was purchased from Roche Diagnostics (Risch-Rotkreuz, Switzerland). Nonfat-dried bovine milk, Fluorescein isothiocyanate-Dextran (FITC-Dextran), Fluorescein isothiocyanate isomer 1, Bovine Serum Albumin, 4-(Dimethylamino)pyridine (DMAP), Pyridine, Succinic Anhydride (SA), 2-(*N*-Morpholino)ethanesulfonic acid (MES), Dimethylmethylene Blue (DMMB), and other salts and reagents were purchased from Sigma (St. Louis, MO, USA). Fetal bovine serum (FBS) was purchased from Atlanta Biologics. Nitric oxide (NO) Griess Reagent System was purchased from Promega (Madison, WI, USA).

### Nucleus pulposus (NP) explant and cartilage ring harvest

NP explants were harvested from adult bovine caudal discs obtained from a local abattoir (Research 87, Boylston, MA, USA). After separating one side of the disc from the vertebra, 1–3 NP explants per disc were extracted using a 6 mm diameter biopsy punch. After harvest, all explants were wrapped in cling film and stored at − 20 °C until use. Cartilage rings were harvested from the condyles of 2-week-old bovine calf knees also obtained from the abattoir. Initially, plugs were extracted from the condyles using an 8 mm diameter biopsy punch and rings were made by using a 6 mm diameter biopsy punch to core the center of the plugs. After harvest, the rings were stored in phosphate buffered saline (PBS) supplemented with protease inhibitors at − 20 °C until use.

### Glycosaminoglycan (GAG) depleted degenerated NP model

GAG depleted NP models were used to simulate early to intermediate-stage degenerative disc disease^40^. In a 24 well plate, NP explants were placed inside cartilage rings to prevent radial swelling and 1 mL of 2% agarose gel was placed around and on top of the cartilage ring-NP setup to maintain NP hydration and mitigate axial swelling. After gel solidification, 1 mL of 1 mg/mL trypsin–EDTA phenol red in PBS was placed on top of the gel for 20 h at 37 °C to cause GAG depletion. GAG content was measured in the explant using the DMMB assay^41^. Explants were rinsed four times in PBS, wrapped in cling film, and stored at − 20 °C until use.

### Selection of solutes

To study the effect of net size and positive charge on the solute transport and retention within the NP tissue, four fluorescently labeled solutes were used (Table 1): (i) fluorescein isothiocyanate isomer 1 (FITC, 390 Da, dia ~ 0.9 nm), (ii) FITC- and Texas Red-conjugated Av (Av, 66 kDa, dia ~ 7 nm), a positively charged globular protein, (iii) FITC- and Texas Red-conjugated Nu (Nu, 60 kDa, dia ~ 7 nm), the electrically neutral complement of Av, and (iv) FITC-Dextran (Dextran, 500 kDa, hydrodynamic dia ~ 16 nm).

**Table 1:** Solute physical properties.

Solute	Diameter (nm)	MW (Da)	Electric charge in 1 × PBS
FITC	0.9	390	Neutral
Nu	7	60,000	Neutral
Av	7	66,000	Positive
Dextran	16	500,000	Neutral

### Transport measurements for solute diffusivities in NP

**Figure 2:**
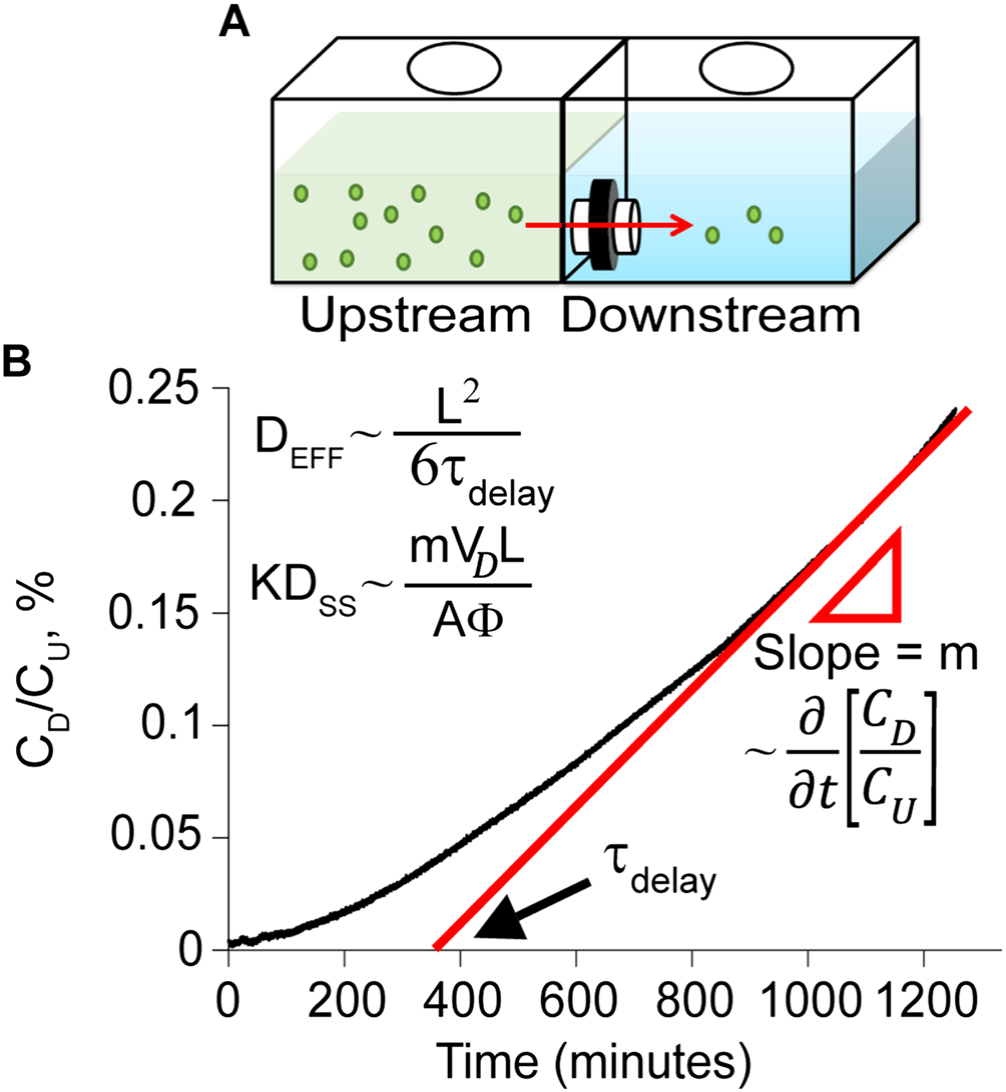
Non-equilibrium diffusion transport of solutes in NP. (**A**) Custom designed transport chamber setup to investigate one-dimensional diffusion of solutes in NP. (**B**) Example diffusion transport curve for Avidin plotted as the measured downstream concentration (C_D_) normalized by the upstream concentration (C_U_), versus time. Intra-NP effective diffusivity, D_EFF_ (diffusion when there are binding interactions) was calculated using ${\uptau }$_delay_ while steady state diffusivity, D_SS_ (diffusion when binding sites are occupied and steady state is reached) was calculated using the steady state slope.

One-dimensional diffusive transport of the FITC labeled solutes through the NP (n = 3 per solute) was measured in real time using a clear poly (methyl methacrylate) transport chamber (Fig. 2A, Supplementary Fig. 1) as previously described^29^. The inner chamber walls were first coated with 0.5% nonfat-dried bovine milk solution in PBS for 15 min to prevent any non-specific binding of solutes to the compartment walls, then rinsed with deionized (DI) water, and dried. The 6 mm diameter NP explants of 2–3 mm thickness were cut using a #10 scalpel blade to a thickness of approximately 1 mm. This explant was then secured by O-rings between the two partitions of the transport chamber with a surface area of 0.1257 cm^2^ exposed for diffusion. The downstream and upstream compartments were each brought to a volume of 2 mL using PBS and allowed to equilibrate. 100 µL of bovine serum albumin was added to the upstream chamber to further coat chamber walls and to promote the stability of solutes, resulting in a final concentration of 0.005% w/v. At t = 0, FITC labeled solutes were added to the upstream chamber for a final upstream concentration of 3 µM while both compartments were kept under constant stirring to inhibit the formation of stagnant layers. A custom-built spectrometer measured the solute concentration in the downstream chamber in real time. 20 µL of upstream solution was transferred to the downstream chamber after the concentration profile in the downstream reached a steady state to determine the upstream concentration at steady state. The effective diffusivity, D_EFF_ (diffusion when there are binding interactions) and steady state diffusivity, D_SS_ (diffusion when binding sites are occupied and steady state is reached) of different sized and charged solutes in NP were extracted from the non-equilibrium diffusion curves.

D_EFF_ was calculated using ${\uptau }_{{{\text{delay}}}}$ which is the time needed to achieve a steady state flux as calculated from the time-axis intercept of the linear slope of normalized concentration versus time. Assuming one-dimensional diffusion of the various solutes through the NP explant of a given thickness, ${\text{L}}$ (~ 1 mm), D_EFF_ can be calculated as^29,42^:

1 $${\text{D}}_{{{\text{EFF}}}} = \frac{{{\text{L}}^{2} }}{{6\tau_{{{\text{delay}}}} }}$$

The axial swelling effect on the thickness of the NP explants was factored into the diffusivity calculations by using the thickness of the explant shortly after steady state at the end of the transport run. Once the steady state diffusion of solutes is reached, the flux (${\Gamma }$) can be correlated to D_SS_ and the concentration gradient across the tissue by the equation^29^:

2 $${\Gamma } = \Phi {\text{KD}}_{{{\text{ss}}}} \frac{{{\text{C}}_{{\text{U}}} - {\text{C}}_{{\text{D}}} }}{{\updelta }} \cong \Phi {\text{KD}}_{{{\text{ss}}}} \frac{{{\text{C}}_{{\text{U}}} }}{{\updelta }},{\kern 1pt} \quad \left( {{\text{as }}\;\;{\text{C}}_{{\text{D}}} \, \ll \,{\text{C}}_{{\text{U}}} } \right)$$

where K is the partition coefficient and ${\Phi }$ is the NP porosity (~ 0.93 measured from wet and dry weights^38^). The time derivative of normalized solute concentration is related to the steady state flux by:

3 $$\frac{\partial }{{\partial {\text{t}}}}\left( {\frac{{{\text{C}}_{{\text{D}}} }}{{{\text{C}}_{{\text{U}}} }}} \right) = \frac{{{\Gamma A}}}{{{\text{V}}_{{\text{D}}} {\text{C}}_{{\text{U}}} }} \cong \frac{{{\Phi{\text KD}}_{{{\text{ss}}}} {\text{A}}}}{{{\delta V}_{{\text{D}}} }}$$

where V_D_ is the volume of the solution in the downstream chamber (V_D_ = 2 mL) and A is the NP surface area exposed to diffusion (A = 0.1257 cm^2^). Using Eqs. 1 and 3, the D_EFF_ and KD_SS_ were estimated for NP tissue.

### Intra-NP retention of solutes

**Figure 3:**
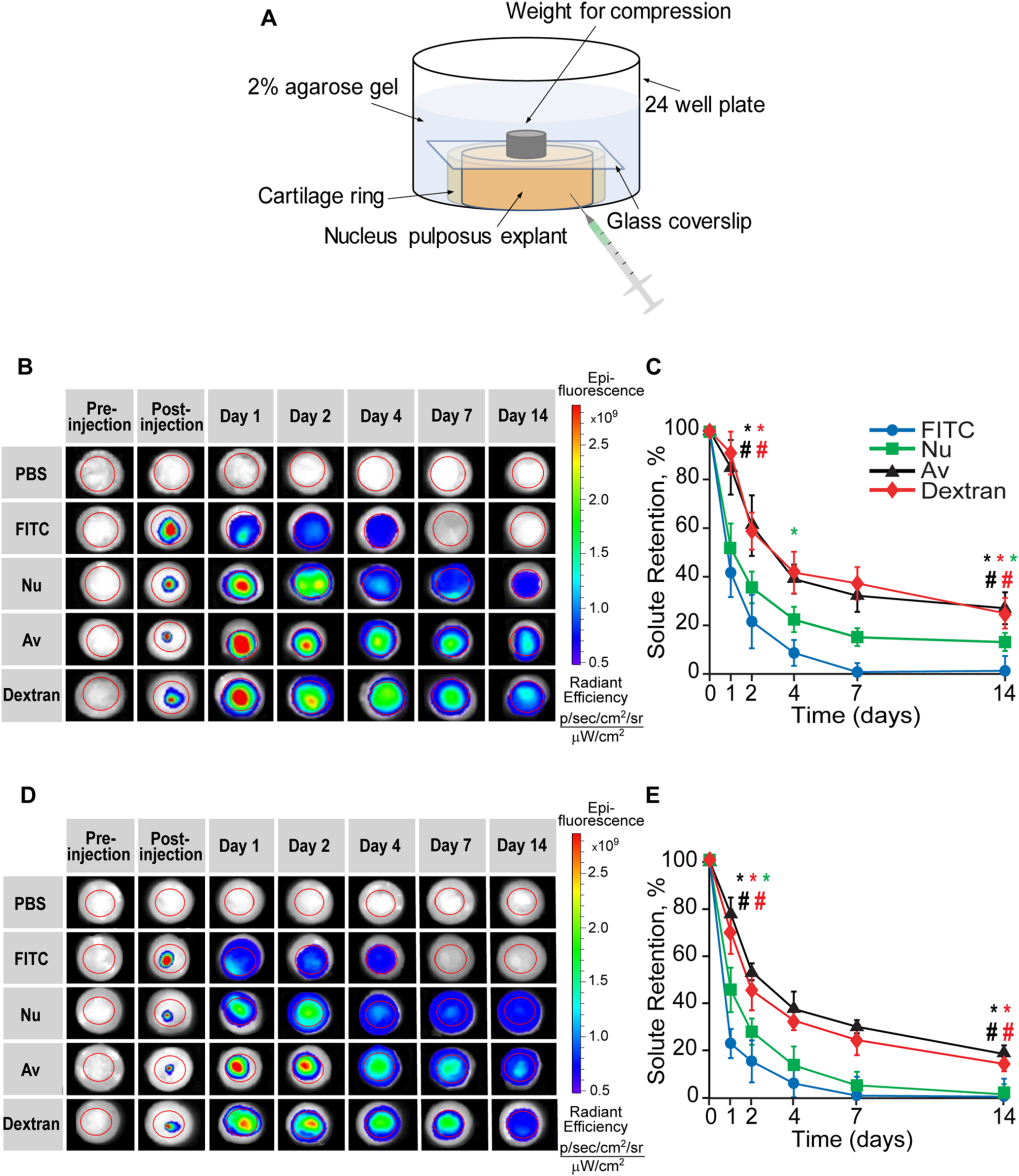
(**A**) IVIS imaging incubation setup to prevent NP swelling while maintaining hydration. (**B**) IVIS panel representing solute retention of FITC (n = 3), fluorescently tagged Neutravidin (Nu) (n = 3), Avidin (Av) (n = 3), and Dextran (n = 3) in healthy NP explants over 2 weeks. (**C**) Intra-NP retention as % solutes remaining in healthy NP over 2 weeks. (**D**) IVIS panel representing solute retention of FITC (n = 3), fluorescently tagged Nu (n = 3), Av (n = 3), and Dextran (n = 3) in degenerated NP explants over 2 weeks. (**E**) Intra-NP retention as % solutes remaining in degenerated NP over 2 weeks (* vs FITC and # vs NeutrAvidin. Statistical markers are color coordinated with all curves. Also, all the data enclosed within the statistical markers are significantly different). Data analyzed using Living Image 4.3 software.

The retention of FITC and Texas Red labeled solutes through the NP was measured over 2–3 weeks using In Vivo Imaging System (IVIS) with 1 s exposure (PerkinElmer, Hopkinton, MA). Healthy (n = 3 per solute) and degenerated (n = 3 per solute) NP explants were placed inside cartilage rings to prevent radial swelling. 2 µL of solutes at concentrations of 30 µM based on the respective moles of conjugated fluorophores were injected into the center of the NP and the time dependent solute diffusion out from the NP center was measured immediately after injection and at day 1, 2, 4, 7, and 14. The acquired images were analyzed using the Living Image 4.3 software to normalize the explants by the noninjected control and to set a consistent fluorescence scale across all solutes and timepoints. The fluorescence overlay and the quantitative fluorescence values from the center of the explant to its edge (a total distance of 3 mm) were extracted. The fluorescence values were further processed in MATLAB R2019a where the area under the curve was integrated to obtain the total fluorescence of each solute at each time point. These values were then normalized by the fluorescence signal of each solute at post-injection and plotted as the percentage of solute retention in the NP over time and the mean of the explants of each solute at each timepoint was taken. In between imaging sessions, the explants secured within the cartilage rings were incubated at 37 °C in a 24 well plate with 2% agarose gel (Fig. 3A). The glass coverslip and weight were needed to prevent axial swelling and to restrict transport to the transverse direction, rather than in the axial direction.

The intradiscal retention half-life (${\uptau }_{{{\text{half}}}}$) of each solute was estimated by fitting an exponential curve to the percent solute retention curves as described by the equation:

4 $${\text{C}}\left( {\text{t}} \right) = {\text{C}}_{0} {\text{e}}^{{ - {\text{t}}/{\uptau }_{{{\text{half}}}} }}$$

where C(t) is the time-dependent percent solute retention and C_0_ is the initial percentage of solutes in the explants at post-injection which was 100% for all conditions. To determine the correlation between NP GAG content and solute retention half-life, the total tissue GAG content was measured using DMMB assay at the conclusion of the 14 day experiment after explant digestion with Proteinase-K ^41^.

### Avidin grafted Dextran branched nanostructure

To combine the effects of positive charge and large size, Av was grafted on Dextran using carbodiimide chemistry. 2 mg of Dextran was dissolved in 300 µL of DMSO. 0.4 mg of SA and 0.6 mg of DMAP were then dissolved in 300 µL of Pyridine which was transferred to the Dextran and DMSO solution. Remaining air was removed using nitrogen before capping the tubes. The reaction was covered from light and left stirring at room temperature for 24 h. After 24 h, the carboxylated Dextran solution was frozen at − 80 °C and lyophilized. The carboxylated Dextran was dissolved in MES buffer for a final concentration of 1 mg/mL. 0.8 mg of EDC and 2 mg of NHS were dissolved in the Dextran and MES solution and reacted for 30 min at room temperature while covered from light. Excess reagents were then removed by dialysis for 1 h in PBS at 4 °C while covered from light. Texas Red conjugated Av in different molar ratios was added to Dextran (10:1, 5:1, 2:1 and 1:1) and reacted for 2 h. Once lyophilized, Av-Dextran was reconstituted in PBS to a concentration of 30 µM of Dextran. The overall charge of Av-Dextran was measured through Zetasizer Nano ZS90 in 1 mg/mL DI water (Malvern Panalytical), and the conjugate with highest zeta value was then evaluated using IVIS for solute retention as previously described.

### Avidin cytotoxicity studies

#### Single dosing NP explant response

NP tissue was harvested under sterile conditions from lumbar disc segments of freshly slaughtered juvenile cows obtained from an abattoir (Research 87, Boylston, MA, USA). 4 mm bovine NP explants were cultured cast in 2% agarose to limit explant swelling and associated inflammatory response in DMEM + 10% FBS + 1% antibiotic/antimycotic^43^. Explants were treated with 0.5 mL of Av in concentrations from 0.1 to 50 µM for 4 days, after which the explants were cultured in base media for up to 15-days total culture (n = 3 per concentration). The explants were treated for only 4 days to prevent the build-up of excess Av due to the charge affinity of Av for the NP GAGs. The explants were collected at the end of 9 days for viability evaluation and at 15 days for biochemical analysis. Live/dead staining was performed with Calcein AM (live)/Ethidium Homodimer-1 (dead). Some explants were digested overnight in papain (0.3 mg/mL papain in 100 mM sodium acetate, 10 mM cysteine HCl, 50 mM EDTA buffer) for biochemical analyses. DNA content was measured using the Pico Green Assay, and explant GAG content using the DMMB assay. Explant water content was also determined by subtracting the dry weight after lyophilization from the wet weight of explants.

#### Avidin dose response in NP cells

NP tissue was harvested under sterile conditions from lumbar disc segments of freshly slaughtered juvenile cows obtained from an abattoir (Research 87, Boylston, MA, USA). NP cells were isolated from tissue via enzymatic digestion (0.3 mg/mL collagenase type I, 0.3 mg/mL collagenase type II) and cultured in complete media: high glucose DMEM + 10% FBS + 1% antibiotic/antimycotic. Cells were expanded (P1–2) prior to use. Bovine NP cells were seeded at 100,000 cells per well in a 96 well plate in high glucose DMEM + 1% antibiotic/antimycotic and treated with Av in concentrations from 0.1 to 50 µM at 37 °C for 4 days (n = 3 per concentration). Supernatants were collected after 24 h and after 4 days for analysis. For each condition, the nitric oxide (NO) release of the NP cells was measured by the Griess Reaction (Promega, G2930) as an indicator of cell inflammatory signaling and cell stress^44^.

### Statistical analysis

All the data presented here is shown as mean ± standard deviation. ANOVA with Fisher LSD post-hoc test was used to test the effect of Avidin concentration on NP explant biochemical outcomes and on explant and cell viability, with p < 0.05 considered significant. Student’s t-test was used to compare treatment conditions over time. A value of p < 0.05 was considered statistically significant.

## Results

### Effect of size and charge on solute transport in NP

The transport chamber setup shown in Fig. 2A was used to study the one-dimensional diffusion of the solutes in the NP. Figure 2B shows a diffusion transport curve for Av plotted as the concentration measured in the downstream chamber (C_D_) normalized by the upstream concentration (C_U_) plotted against time. Effective diffusivity, D_EFF_, and steady state diffusivity, D_SS_, were calculated as described in “Materials and methods”.

Table 2 shows the diffusivity estimates of solutes in healthy NP tissue. D_EFF_ for Dextran was an order of magnitude lower than that for Nu or FITC due to steric hindrance, mediated by its large molecular weight (p < 0.05). Moreover, D_EFF_ for Av was an order of magnitude lower than that for Nu due to electrostatic interactions. D_EFF_ for Av was found to be two orders of magnitude slower than KD_SS_. Nu, which is the same size as Av but is electrically neutral, did not exhibit a significant difference between D_EFF_ and KD_SS._ These differential behaviors are due to electrostatic binding of Av with intra-NP proteoglycans. Dextran also showed no significant difference between its D_EFF_ and KD_SS_ confirming slow diffusion is due to steric hindrance and not binding interactions. Additionally, FITC exhibited one order of magnitude difference between its D_EFF_ and KD_SS_ that can be attributed to the hydrophobic nature of dye that can lead to non-specific binding within the hydrophobic tissue pockets (p < 0.05). The partitioning factor, K, for FITC and Nu is estimated to be 1 as they are uncharged molecules and small enough to easily diffuse through the NP pores without much steric hindrance. K for Av is estimated by calculating the ratio of (KD_SS_)_Av_ to (KD_SS_)_Nu_ which is equal to 4. Av and Nu are of similar size and, thus, are expected to have similar D_SS_. Since K_Nu_ is estimated to be 1, K_Av_ is calculated as ~ 4. Due to the large size of Dextran (16 nm diameter), its partitioning is expected to be less than 1.

**Table 2:** Effective (D_EFF_) and steady state (D_SS_) diffusivities in healthy NP tissue.

Solute	D_EFF_ (cm^2^/s)	KD_SS_ (cm^2^/s)	K
FITC	$2.1 \pm 0.2 \times 10^{ - 6}$	$6.6 \pm 1.4 \times 10^{ - 5}$	~ 1
Nu	$3.6 \pm 3.3 \times 10^{ - 6}$	$6.0 \pm 2.8 \times 10^{ - 6}$	~ 1
Av	$4.1 \pm 2.7 \times 10^{ - 7}$	$2.4 \pm 2.2 \times 10^{ - 5}$	~ 4
Dextran	$1.2 \pm 0.3 \times 10^{ - 7}$	$4.5 \pm 3.0 \times 10^{ - 7}$	< 1

Table 3 shows the diffusivity estimates of solutes in early to intermediate-stage degenerated NP tissue. Using DMMB assay, we confirmed that degenerated samples exhibited a 44.7 ± 17.3% loss in GAG content compared to normal samples (Table 4). This is consistent with early or intermediate degeneration which is the ideal candidate for a drug delivery intervention^9^. D_EFF_ for Av remained two orders of magnitude slower than KD_SS_ despite approximately 45% less GAGs in degenerated NP explants indicating that Av can bind with the remaining GAGs due to the long-range effects of charge interactions. Overall, D_EFF_ and KD_SS_ of Av in degenerated explants remained similar to healthy NP. Dextran, however, showed significantly (2 ×) faster transport in degenerated explants compared to healthy due to a larger pore size in the GAG depleted explants (p < 0.05). K for FITC, Nu, and Dextran was estimated to be close to 1 due to reduced steric hindrances in the GAG depleted explants and no electrostatic binding. K for Av remained unchanged at about 4.

**Table 3:** Effective (D_EFF_) and steady state (D_SS_) diffusivities in degenerated (50% GAG depleted) NP tissue.

Solute	D_EFF_ (cm^2^/s)	KD_SS_ (cm^2^/s)	K
FITC	$1.8 \pm 0.7 \times 10^{ - 6}$	$4.8 \pm 3.9 \times 10^{ - 5}$	1
Nu	$2.5 \pm 1.0 \times 10^{ - 6}$	$7.8 \pm 1.9 \times 10^{ - 6}$	1
Av	$3.2 \pm 2.5 \times 10^{ - 7}$	$3.4 \pm 0.4 \times 10^{ - 5}$	~ 4
Dextran	$2.1 \pm 0.6 \times 10^{ - 7}$	$8.9 \pm 0.8 \times 10^{ - 7}$	1

**Table 4:** GAG content of healthy and degenerated NP explants.

Tissue	GAG Content (µg/mg)
Healthy	$37.2 \pm 4.9$
Degenerated	$20.6 \pm 6.4$

### Effect of net size and charge on solute intra-NP retention

Figures 3B,D show representative images of solutes retained in the NP explant after intra-NP injection. Solute content (FITC, Nu, Av, or Dextran) was tracked using IVIS over 14 days, and retention was quantified as % relative to day 0. The smallest solute in this study, FITC, diffused out rapidly from both healthy and degenerated NP; by day 1, only 40% of FITC retained inside healthy NP which was significantly reduced to 20% retention in degenerated NP (p < 0.05, Fig. 3C,E). No traces of FITC were observed at 7 and 14 days in healthy and degenerated NP respectively. Higher percentage of Nu was retained in healthy NP when compared to FITC on day 4, 7, and 14 (p < 0.05), but showed no difference to FITC in degenerated NP after 24 h (Fig. 3C,E). Furthermore, Av and Dextran had significantly higher retention in both healthy and degenerated NP compared to FITC and Nu on all days (p < 0.05) (Fig. 3C,E).

Table 5 shows the intra-NP retention half-life of solutes in healthy and degenerated NP. Both FITC and Nu have a retention half-life of about 1 day and Nu’s retention half-life dropped significantly in degenerated NP by 1.6 × (p < 0.05). Av and Dextran retained within healthy NP until 14 days measuring a retention half-life of approximately 3 days while both Nu and FITC had a 3 × shorter retention half-life. Dextran’s retention half-life dropped significantly to 1.9 days in degenerated NP (p < 0.05) while Av’s retention half-life remained unchanged owing to electrostatic binding.

**Table 5:** Intra-NP diffusion properties and retention half-lives of solutes in healthy and degenerated NP.

${{\varvec{\uptau}}}_{{{\mathbf{half}}}}$(days)	FITC	Nu	Av	Dextran	Av-Dextran
Healthy	$0.9 \pm 0.2$	$1.3 \pm 0.2$	$2.7 \pm 0.6$	$3.0 \pm 0.4$	$6.5 \pm 0.8$
Degenerated	$0.7 \pm 0.3$	$0.8 \pm 0.1$	$2.6 \pm 0.6$	$1.9 \pm 0.4$	$4.8 \pm 0.8$

### Combined effects of solute size and cationic charge on intra-NP transport and retention

**Figure 4:**
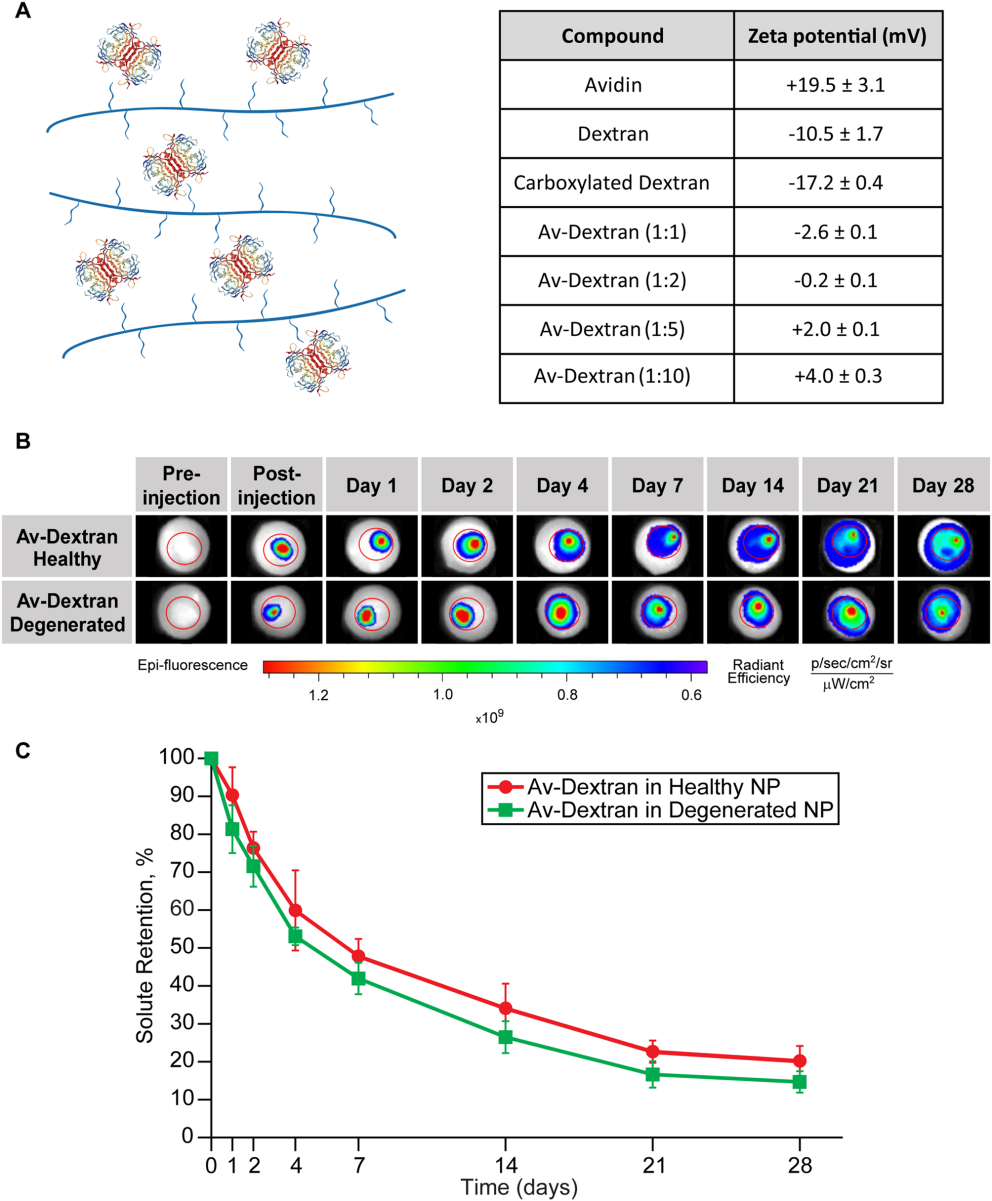
(**A**) Schematic representation of Avidin grafted Dextran (Av-Dextran) nano-construct along with the zeta potential values of Av, Dextran, carboxylated Dextran and Av-Dextran at different molar ratios. (**B**) IVIS panel with both healthy (n = 3) and degenerated NP (n = 3) showing retention of Avidin-Dextran over 28 days. (**C**) Quantitative estimate of intra-NP retention of Avidin–Dextran in healthy and degenerated NP over 4 weeks. Data analyzed using Living Image 4.3 software.

Schematic of Av grafted Dextran (Av-Dextran) constructs along with the zeta potential values of Av, Dextran and Av-Dextran in different molar ratios are shown in Fig. 4A. To study the combined effects of size and charge, Av-Dextran structure with the highest zeta potential value (or cationic charge) was used. Using the same transport setup shown in Fig. 2A, one-dimensional diffusion properties (D_EFF_ and D_SS_) in both healthy and degenerated NP were measured (Table 6). D_EFF_ for Av-Dextran was two orders of magnitude slower than D_SS_ again confirming electrostatic binding of Av-Dextran with intra-NP proteoglycans. Furthermore, D_EFF_ of Av-Dextran is one order of magnitude slower than both Av and Dextran, which can be explained by the combined effect of electrostatic interactions and increased steric hindrance. Diffusion of Av-Dextran was measured to be slightly faster (but not statistically significant) in degenerated NP than in healthy NP likely due to the loss of GAGs and larger pore size.

**Table 6:** Effective (D_EFF_) and steady state (D_SS_) diffusivities of Av-Dextran in healthy and degenerated NP.

Av-Dextran	D_EFF_ (cm^2^/s)	KD_SS_ (cm^2^/s)
Healthy	$5.8 \pm 3.2 \times 10^{ - 8}$	$2.7 \pm 1.1 \times 10^{ - 6}$
Degenerated	$6.5 \pm 3.5 \times 10^{ - 8}$	$4.0 \pm 2.3 \times 10^{ - 6}$

Figure 4B shows the IVIS results of Av-Dextran in both healthy and degenerated NP over 28 days and Fig. 4C shows the solute retention of Av-Dextran in healthy and GAG depleted NP. Av-Dextran retained within both healthy and degenerated NP for the entire 28 days reaching a retention half-life of nearly 1 week in healthy NP and 5 days in degenerated NP (Table 5). Av-Dextran had over a 2 × longer retention half-life than Av and Dextran in both healthy and degenerated NP which was statistically significant (p < 0.05) and can be attributed to the combined effects of charge interactions and large solute size.

### Effect of NP GAG content on intra-NP retention half-life

**Figure 5:**
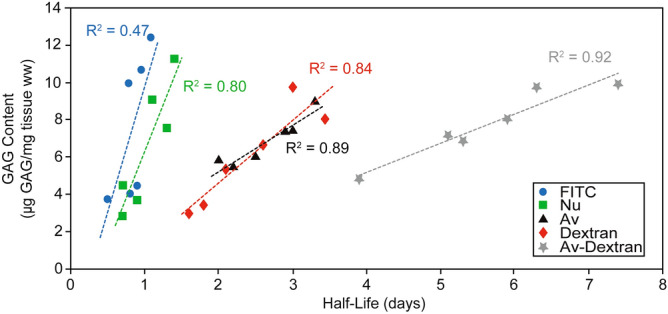
Correlation between NP GAG content and the intra-NP retention half-lives of different solutes. NP GAG content is normalized by the explant wet weight (WW).

To examine the relationship between GAG content and solute retention, we compared solute half-life across both control and GAG-depleted samples. As expected, we found that the solute retention half-life decreased with decreasing GAG content (Fig. 5). Both FITC and Nu show a smaller dependence of retention half-life based on GAG content compared to Av and Dextran, as indicated by the lower slope. Furthermore, the correlation between GAG content and retention half-life is low for FITC indicating that the retention half-life of FITC is not dependent on NP GAG content as FITC is not positively charged and is small enough to easily diffuse out of both healthy and degenerated NP. The depletion of GAGs reduces the net fixed charge density and steric hindrances in NP. Consequently, both Av and Dextran show a much stronger relationship between GAG content and retention half-life. This correlation was stronger for Av-Dextran further confirming the combined effects of size and charge in enhancing intra-NP retention.

### Dose dependent biological response of Av on NP health

**Figure 6:**
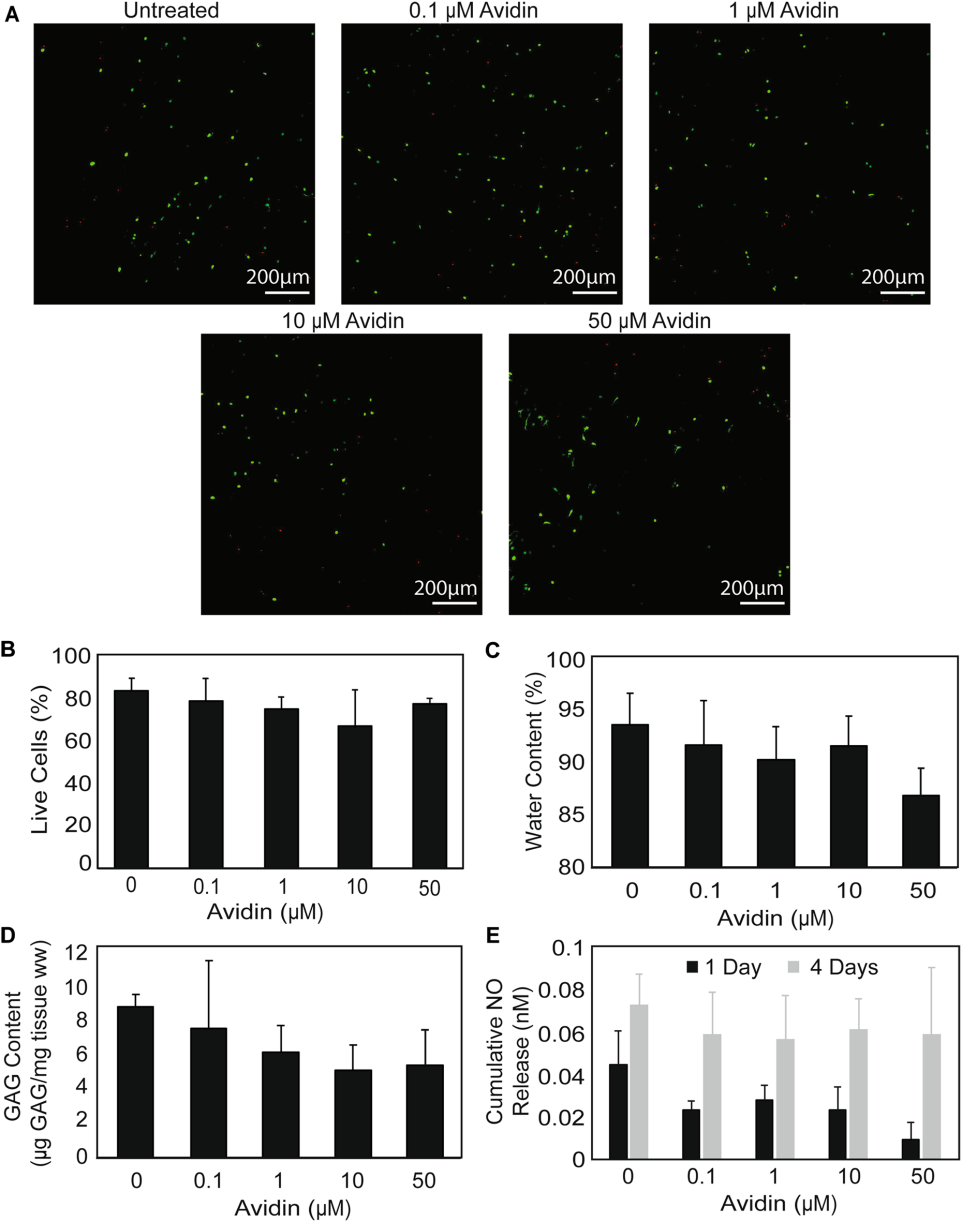
Dose dependent biological response of Avidin on NP. (**A**) Live/dead staining of NP explants after 9 days of culture under different concentrations of Avidin (n = 3). (**B**) Percentage of live cells in NP explants after 9 days of culture with increasing concentrations of Avidin (n = 3). (**C**) Water content of NP explants after 15 days of culture under different concentrations of Avidin (n = 3). (**D**) Residual GAG remaining in NP explants after 15 days of culture with different concentrations of Avidin (n = 3). GAG content represented as µg GAG normalized by explant wet weight (WW). No statistical difference between the Avidin treated conditions and control was noted. (**E**) Cumulative NO release from NP cells under culture with increasing concentrations of Avidin (n = 3).

Cell viability in NP explants cultured in the presence of Av from 0.1 to 50 µM over 9 days showed no significant difference between Av treated groups and untreated control, indicating that Av does not induce cell death even at high doses of 50 µM (Fig. 6A,B). Both the NP explant GAG content per wet weight (WW) and NP water content were not affected with Av treatment over 15 days of culture, indicating that Av does not have a negative effect on NP GAG content or water content (Fig. 6C,D). Furthermore, the nitric oxide release of NP cells cultured in the presence of Av from 0.1 to 50 µM over both 1 and 4 days showed no significant difference between Av treated groups and untreated control, indicating that Av did not promote inflammatory or cell stress responses (Fig. 6E). This suggests that Av could be safely used as a drug carrier for targeting NP.

## Discussion

The goal of this study is to develop drug delivery systems that are capable of prolonged intra-discal retention to provide a longer lasting therapeutic benefit. We show that that the high negative fixed charge density of NP can be used to enhance residence time by using cationic carriers that can create intra-discal depots to enable sustained therapeutic benefits with a single dose injection. Using Av grafted Dextran nanostructures, we demonstrate synergistic effects of cationic charge and larger carrier size to sufficiently slow down intra-NP diffusivities due to electrostatic binding and steric hindrances enabling a month long intra-NP residence time following intra-discal administration. Extended residence time was also observed in GAG-depleted samples that mimic early to intermediate disc degeneration^9^. The cationic charge conferred by Av to the conjugate is optimal to enable its binding with intra-NP GAGs which is strong enough to prevent rapid diffusion out from the injection site but weak enough so Av functionalized Dextran can disperse through the full NP explant, which is important to reach target cells throughout the tissue. We further identified a relationship between GAG content and solute retention half-life, conferring additive effects of steric hinderance (due to MW) and electrostatic charges.

Real-time transport data using custom transport chambers (Fig. 2) shows that the effective intra-NP diffusivity of Av is slower than its neutral counterpart, Nu, owing to charge-based binding. Its effective diffusivity (D_EFF_) was two orders of magnitude slower than its steady state diffusivity (D_SS_) further confirming its binding with intra-NP proteoglycans (Table 2). Diffusivity did not change in degenerated NP (Table 3) indicating that Av can effectively interact with the remaining negatively charged GAGs via long-range effect of charge interactions. Dextran also exhibited slower diffusivities compared to Nu and FITC due to its large size. No significant difference between its effective and steady state diffusivities was found further confirming that its slow diffusion is attributed to steric hindrance, not binding effects. Its diffusivity, however, increased by 2 × in degenerated explants compared to healthy (Table 3), further demonstrating the role of steric hinderance for dextran diffusion. These findings were also corroborated by IVIS imaging data (Fig. 3) which showed an intra-NP mean half-life of about 2.7 days for Av in both healthy and degenerated tissues and of about 3 days for Dextran in healthy tissue which was reduced to 1.9 days in degenerated tissue (Table 5). The majority of FITC (which is similar in size to a small molecule drug), diffused out rapidly from both healthy and degenerated NP following injection. Our data highlights that while increasing carrier size can enhance retention in healthy NP, degenerated tissues with increased pore size require long-range binding effects to enhance retention which we achieve via electrostatic interactions.

Av-Dextran exhibited an order of magnitude slower diffusivity compared to Av or Dextran alone; its effective diffusivity was two orders of magnitude slower than its steady state diffusivity confirming slowed diffusion due to binding with intra-NP sites (Table 6). Furthermore, Av-Dextran retained within both healthy and degenerated NP for the entire 28 days reaching a retention half-life of nearly 1 week in healthy NP and 5 days in degenerated NP (Table 5), which is two times longer than Av or Dextran alone. Av-Dextran half-lives exhibited a strong positive correlation with tissue GAG content.

Av is a tetrameric protein rich in arginine and lysine with high pI of 10.5, and its binding with aggrecan-GAGs is largely attributed to charge interactions^29^ but other non-specific interactions are also at play. It was recently shown that long-range charge-based binding can be stabilized by short-range H-bonds and hydrophobic interactions mediated by arginine residues in protein structure^36^. The guanidinium head-group on arginine forms stable bidentate hydrogen bonds with sulfates in aggrecan GAGs. Additionally, guanidinium cations can form thermodynamically stable (weakly) like-charge pairs in water enabling arginine rich sites on Av to interact cooperatively and bind strongly with GAGs. This is especially relevant for targeting mid to late stage degenerated tissues that have lost some GAGs and have lower negative fixed charge density^36^.

IVD degeneration is mediated by several endogenous damage-associated molecule patterns like ECM fragments or pro-inflammatory cytokines^45,46^. Blocking of inflammatory signaling via small molecule inhibitors (e.g. dexamethasone) or large neutralizing cytokine antibodies are popular approaches for mitigation of inflammatory signaling in DD^45,47–49^. However, both types of small and large molecule drugs have limited retention in the disc. For example, a recent study reported that a single dose intra-discal injection of glucocorticoid in patients with LBP reduced pain within 1 month, however, was ineffective at the 12 month time point^13^. A combination therapy including treatment with a pro-anabolic growth factor like GDF-5 to regenerate tissue matrix shifting the cell homeostasis to the anabolic state has also been proposed^50^. Strong preclinical animal data has resulted in two ongoing multicenter clinical trials evaluating safety and efficacy of a single intra-discal GDF-5 injection for early treatment of lumbar disc degeneration (NCT00813813)^51^ but concerns around limited efficacy and associated toxicity remain. It is, therefore, key to incorporate drug delivery approaches at early stages of drug development. Av grafted dextran nanostructures provide multiple sites for direct conjugation of multiple drugs via controlled release linkers enabling combination therapy. Av and Av mimicking peptides can also be functionalized on the surfaces of drug encapsulating micron and nanoparticles or directly conjugated to or supramolecularly combined with larger sized protein drugs, antibodies or nucleic acid to prolong their intra-discal residence time to a month period.

Recently, tissue regeneration using progenitor cell-based approaches including cell-seeded hydrogels have gained attention for IVD repair^52,53^. However, the harsh environment of the degenerated disc may significantly affect differentiation capability of the progenitor cells^52^. Therefore, effective delivery of anti-inflammatory and anti-catabolic therapeutics is necessary to mitigate the harsh microenvironment of the disc for successful tissue regeneration^54^. Several drug delivery systems have been developed for sustained delivery of therapeutics in IVDs^18,54,55^. For example, PLGA microspheres encapsulating interleukin-1 receptor antagonist maintained drug release for 20 days, thereby attenuating the degradative effects of interleukin-1 beta^18^. When injected in the caudal discs of Sprague–Dawley rats, these microspheres were detectable in the discs up to 28 days post injection^55^. However, at this time point, for 63% of the discs, the microspheres were localized in the annulus fibrosus only (no presence in the NP) while in only 13% of the discs the microspheres were still retained completely in the NP section, suggesting significant diffusion of the microsphere out of the NP^55^. Therefore, despite providing a sustained drug release, these systems fail to maintain localized retention of the drug after its release, allowing these biologics to diffuse out of the IVD, thereby decreasing efficacy of the biological response and possibly affecting the function of surrounding tissues through wide spreading. Further, modification of chymopapain loaded pluronic nanocarriers with cationic chitosan polymers has shown to significantly enhance NP localization of the nanocarriers 24 h post injection in the discs, underlying the significant role of electrostatic interactions with the negatively charged NP GAGs^24^. Moreover, positively charged cysteine-dense peptides with stable conformations consisting of cysteine-knot folds were shown to accumulate in mice IVDs and knee cartilage post intravenous injection owing to electrostatic interactions with matrix negatively charged proteoglycans^57^. Additionally, cysteine dense peptide-triamcinolone acetonide conjugate showed 33 times higher accumulation in the IVDs compared to the free triamcinolone acetonide drug 24 h after systemic administration, showing potential of using electrostatic interactions for targeted drug delivery to IVDs^57^. The Avidin-Dextran complex, taking advantage of synergistic effects of positive charge and larger size, provides over 1 month intra-NP retention through combining electrostatic interactions and steric hinderance. Therefore, when paired with therapeutic agents, Avidin-Dextran can potentially maintain intra-NP localized retention enabling an effective biological response while leading to less complications by preventing spreading of the therapeutic agents outside of the IVD.

## Conclusion

We demonstrate cationic Av grafted Dextran structures can increase intra-NP residence times to 1 month, significantly longer than previously shown for any carrier material. The carrier can be used for delivering a variety of drugs and their combination to cells within NP and reduce or eliminate the need for multiple injections of high drug doses and minimize associated side-effects. Av can also be functionalized on the surface of other larger sized drug encapsulating particles. This work can pave way for effective clinical translation of potential therapeutics for treatment of LBP and disc degeneration.

## Supplementary information

Supplementary Information 1.

Supplementary Information 2.
