# Supplementary figures and images for: Avidin grafted dextran nanostructure enables a month-long intra-discal retention
Source: Sci Rep. 2020 Jul 21;10:12017. doi: 10.1038/s41598-020-68351-1 (PMC7374582; doi:10.1038/s41598-020-68351-1)

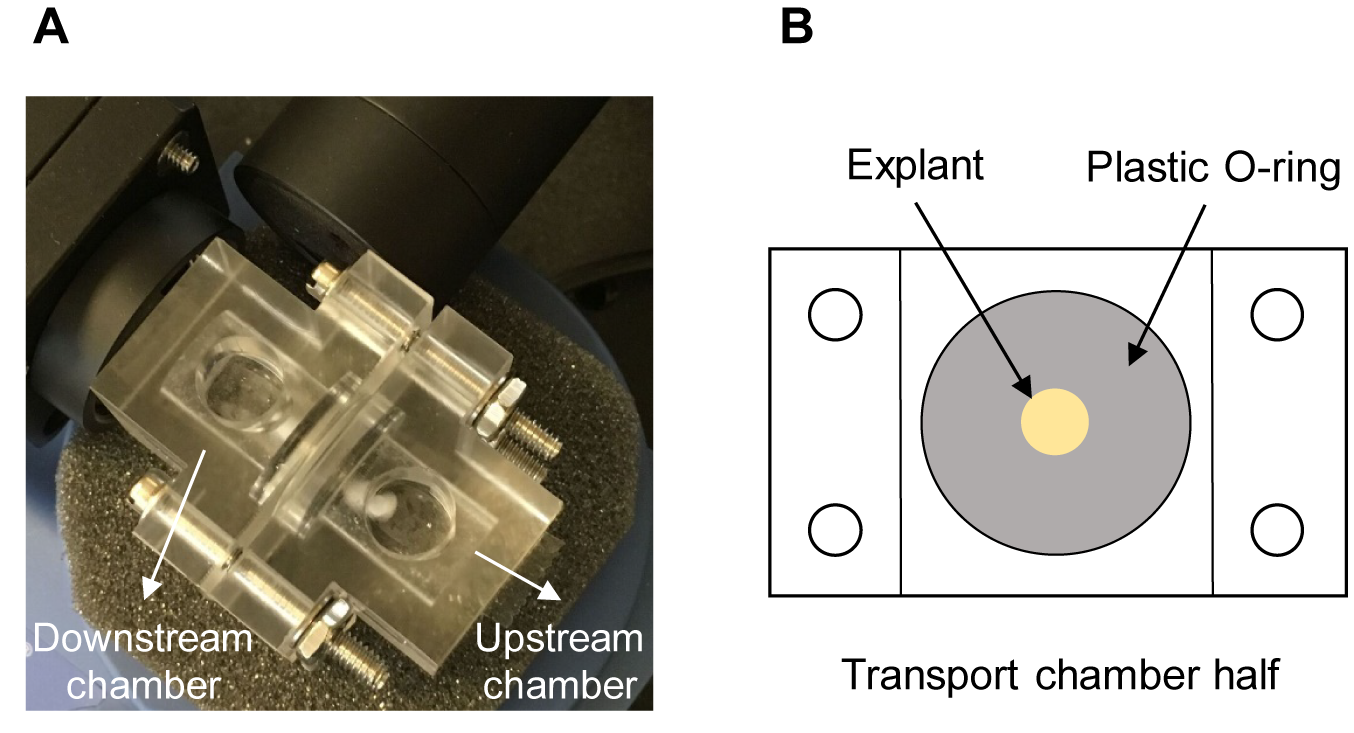

Supplement: Supplementary file 1 — Supplementary Information 1. [file 41598_2020_68351_MOESM1_ESM.tif]
