# Supplementary material for: Avidin grafted dextran nanostructure enables a month-long intra-discal retention
Source: Sci Rep. 2020 Jul 21;10:12017. doi: 10.1038/s41598-020-68351-1 (PMC7374582; doi:10.1038/s41598-020-68351-1)
Supplement: Supplementary file 2 — Supplementary Information 2. [file 41598_2020_68351_MOESM2_ESM.docx]

**Supplementary Figure Captions**

**Supplementary Figure 1.** Custom designed 1-D non-equilibrium diffusion transport chamber setup. **A.** Transport chamber setup consisting of two identical poly (methyl methacrylate) half chambers sandwiched together with the NP explant in the middle. The setup allows 1-D diffusion of fluorescently labeled solutes from the upstream chamber to the downstream chamber. The solution in the downstream is excited using an appropriate laser line matching the excitation wavelength of the fluorescent dye. **B.** Cross-sectional view of the transport chamber half showing the plastic O-ring that is used for placing the explant and loading on the transport chamber half.
